# The role of the sulcus angle in patellar dislocation: The importance of measuring four magnetic resonance imaging axial levels and utilising corresponding cutoff values

**DOI:** 10.1002/jeo2.70425

**Published:** 2025-09-05

**Authors:** Jason D. Brenner, Steven M. Henick, Leila Mehraban Alvandi, Edina Gjonbalaj, Yungtai Lo, Jacob Schulz, Eric D. Fornari, Benjamin J. Levy, Mauricio Drummond Junior

**Affiliations:** ^1^ Albert Einstein College of Medicine The Bronx New York USA; ^2^ Department of Orthopaedic Surgery Montefiore Einstein The Bronx New York USA

**Keywords:** cutoff value, MRI measurements, patellar instability, sulcus angle, trochlear dysplasia

## Abstract

**Purpose:**

The primary purpose was to assess sulcus angle (SA) magnetic resonance imaging (MRI) measurements and determine diagnostic cutoff values along four axial levels on cartilaginous and osseous surfaces comparing those with patellar dislocations (PD) versus controls. A secondary aim was to identify differences in SA between patients with one‐time dislocation (OTD) versus recurrent patellar dislocations (RPDs).

**Methods:**

Paediatric patients with a history of PD were retrospectively grouped into those with an OTD versus RPDs. Age and sex frequency matching controls (ACL injuries without PD history) were identified. The SA was recorded at four levels in the trochlear groove (TG) on cartilaginous and osseous surfaces. Differences between sample means (PDs vs. controls; RPDs vs. OTDs) were assessed; cutoff values for discriminating PDs from controls were identified utilising Youden′s index.

**Results:**

There were 173 PDs (106 RPDs, 67 OTDs) and 100 controls. There were differences in mean SA between PD and controls throughout the trochlear groove for both cartilaginous (PD vs. control: SA1 166.1° vs. 152.5°, SA2 161.0° vs. 148.5°, SA3 155.7° vs. 145.9°, SA4 150.7° vs. 142.5°) and osseous surfaces (PD vs. control: SA1 160.2° vs. 146.6, SA2 153.8° vs. 140.2°, SA3 147.2° vs. 134.8°, SA4 142.1° vs. 132.6°) (*p* < 0.001). Diagnostic cutoffs were higher for cartilaginous versus osseous measurements (SA1 159.6° vs. 153.1°, SA2 153.8° vs. 148.0°, SA3 152.5° vs. 141.6°, SA4 148.1° vs. 137.4°). RPD patients had greater cartilaginous SA than OTDs throughout the TG (SA1*p* = 0.014, SA2*p* = 0.004, SA3*p* = 0.027, SA4*p* = 0.007), while osseous SA measurements did not differ (SA1*p* = 0.057, SA2*p* = 0.070, SA3*p* = 0.185, SA4*p* = 0.175).

**Conclusions:**

SA was greater in PDs than controls at all four levels in the TG for both cartilaginous and osseous measurements. Cartilaginous SA was greater among RPDs than OTDs at all levels; however, osseous SA was not different between cohorts. The diagnostic cutoff of dysplastic SA differed by axial level and surface.

**Level of Evidence:**

Level III.

**Abbreviations**
(c): cartilaginous
(o): osseous
ACL: anterior cruciate ligament
AUC: area under the curve
CI: confidence interval
ICC: intraclass correlation coefficient
IQR: interquartile range
MPFL: medial patellofemoral ligament
MRI: magnetic resonance imaging
OTD: one‐time dislocation/dislocator
PACS: picture archiving and communication system
PD: patellar dislocation/dislocator
PI: patellar instability
PT: patellar tilt
ROC: receiver operating characteristics
RPD: recurrent patellar dislocation/dislocator
SA: sulcus angle
SPSS: Statistical Package for the Social Sciences
TD: trochlear dysplasia
TT‐TG: tibial tubercle‐trochlear groove

## INTRODUCTION

Patellar dislocation (PD) is a common and debilitating paediatric knee injury with an estimated incidence of 23.3–49 per 100,000 person years [9, 22, 25, 27, 30]. The aetiology of PD is multifaceted and associated with excessive femoral anteversion, lateral patellar tilt (PT), increased tibial tubercle to trochlear groove (TT‐TG) distance, patella alta, knee rotation, ligamentous laxity, and weak medial soft tissue stabilisers among others [2, 3, 4]. Of these risk factors, trochlear dysplasia (TD) is often considered the key anatomical risk factor (ARF) for both initial and recurrent patellar dislocations (RPDs) [6].

Quantitative radiological measurements of TD are preferred due to its varying severity and morphology [13, 21, 23, 31, 32]. While there are dozens of described methods to assess TD, the sulcus angle (SA) has emerged as a widely adopted technique [2, 7, 17, 29]. SA is formed between the lateral and medial trochlear facets at the distal femur which represents the groove's ‘shallowness’ [34]. Initial ratings of SA were described using plain radiographs; however, this imaging modality does not accurately represent the extent of TD [8, 24]. These radiographs are represented by axial patellofemoral view images which can capture the distal trochlear groove (TG), although it is the proximal TG that is considered more clinically relevant in assessing for TD [20, 24]. The proximal TG represents the shallowest portion and is where the patella initially articulates with the TG during flexion. While the medial patellofemoral ligament (MPFL) is the primary stabiliser from 0° to 30° of flexion, abnormal patellofemoral articulation starting at 30° of flexion is highly correlated with PD due to TD [11].

Magnetic resonance imaging (MRI) is the preferred imaging modality due to its superior resolution for cartilaginous and soft tissue structures, as well as its enhanced ability to detect osteochondral fractures [5, 18, 26, 28, 33]. As such, MRI is considered the gold standard for measuring SA. However, previous studies have assessed SA using only a single axial level with no consensus on the optimal level [1, 19, 31, 36]. Given that the trochlear length is approximately 2 cm from its most proximal to distal extent and that MRIs capture axial images at 3 mm intervals, there are at least four relevant axial levels to record SA and map the TG [4, 35]. Thus, the primary purpose was to assess SA MRI measurements and determine diagnostic cutoff values along four axial levels on cartilaginous and osseous surfaces comparing those with PD versus controls. A secondary aim was to identify differences in SA between patients with one‐time dislocation (OTD) versus RPDs. The authors hypothesised that measuring the SA in different axial levels will yield different optimal cutoff values and that these diagnostic cutoffs will differ between cartilaginous and osseous surfaces [29].

## METHODS

Following approval from the institutional review board (IRB: 2016‐6469), a comprehensive list of patients aged 9–21 at the time of their MRI was compiled from a single institution. These patients were treated for patellar instability (PI) by the Department of Paediatric Orthopaedic Surgery between 2012 and 2023. A retrospective review of medical records was conducted for 230 patients to confirm a history of PD and categorise them into two groups: those who experienced a OTD and those with RPDs, defined as at least two dislocations. Additionally, based on the patients' ages at the dates of MRI, 100 age‐ and sex frequency matching control patients who received MRIs for ACL injuries (and no PD history confirmed by chart review) were recruited from an internal database. Patient classification in the RPD group was confirmed through a review of medical records or telephone follow‐up. Patients were excluded if their MRI scans had insufficient image quality (defined as poor resolution at one or more axial levels of the TG that hindered accurate SA measurement) or if they had undergone prior surgery on the affected knee. To verify the absence of additional PDs, telephone follow‐up was conducted for patients in the OTD group. A minimum follow‐up period of two years from the initial presentation was required.

**Figure 1:**
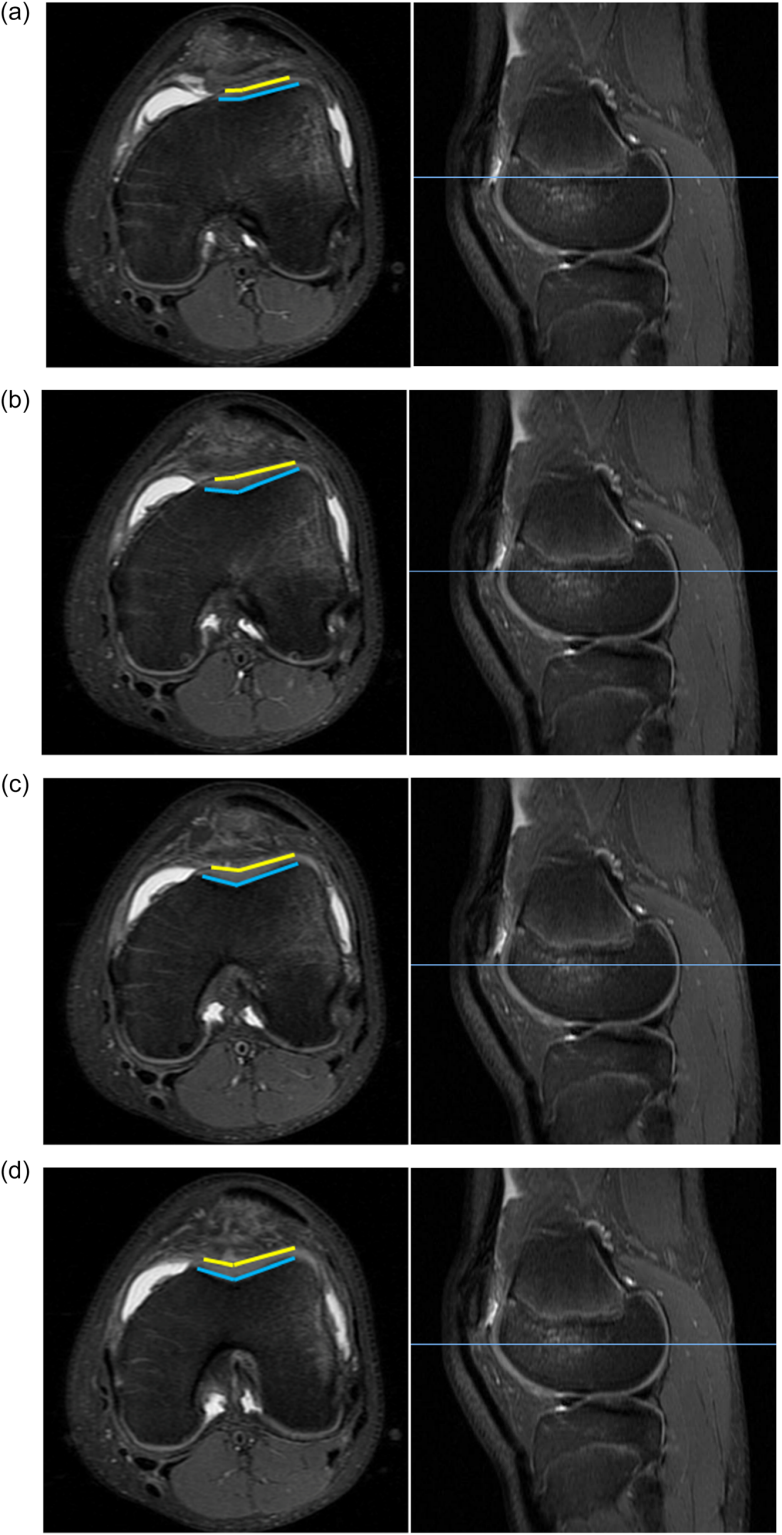
The measurement of (a) proximal SA (SA1), (b) near‐proximal SA, (c) near‐distal SA, (d) distal SA was recorded on T2 weighted MRI. The SA was formed by lines connecting the most prominent portions of the medial and lateral condyles with the deepest point in the TG [10, 15, 29]. The cartilaginous SA was depicted in yellow, and the osseous SA in blue. Created utilising PACS software and Microsoft Word. MRI, magnetic resonance imaging; SA, sulcus angle; TG, trochlear groove.

SA was measured on cartilaginous and osseous surfaces using the method described by Davies et al. [7]. SA was measured at four consecutive axial sequences of the TG: proximal (SA1; Figure 1a), near‐proximal (SA2; Figure 1b), near‐distal (SA3; Figure 1c), and distal (SA4; Figure 1d).

The measurements were conducted independently by a second‐year medical student and a second‐year orthopaedic surgery resident following a video‐recorded training session led by the senior author. All patient MRIs were conducted at a single institution with the knee imaged in the AP plane while in a standard supine position. A 16‐channel knee coil was used to acquire the following sequences: coronal T1‐weighted, coronal proton density fat‐saturated, sagittal proton density, sagittal proton density fat‐saturated, and axial proton density fat‐saturated. The proximal TG was defined as the most superior axial image in which the cartilaginous portions of both the medial and lateral trochlear facets were visible. This slice was cross‐referenced with the sagittal plane for accuracy. This image was used to record cartilaginous SA1 as seen with the yellow angle in Figure 1a; the subsequent three distal axial levels were used to measure cartilaginous SA2–4 (yellow angles in Figure 1b–d). This process was repeated for osseous SA measurements using the subchondral bone immediately posterior to the cartilaginous layer. The same four levels used for the cartilaginous SA1–4 were corresponded and used for osseous SA1–4 and shown in Figure 1a–d with the blue angles.

The normality assumption for all SA variables were evaluated utilising the modified Kolmogorov–Smirnov test. SA variables were reported as means and standard deviations, medians and IQRs, and ranges. The data were graphed as a simple boxplot to visualise the distributions of control versus PD and RPD versus OTD to assess the overlap between interquartile ranges (IQRs). The comparisons with minimal overlap of the IQRs (PD vs. control) were utilised to establish cutoff values; the comparisons of RPD versus OTD were not utilised to establish cutoff values as the overlap in IQR between cohorts would make establishing a meaningful cutoff value difficult.

Statistical analyses were conducted utilising IBM Statistical Package for the Social Sciences (SPSS) for Mac Version 20 (IBM Corp). Independent *t*‐tests were conducted to assess differences in sample means between PDs and controls and between RPDs and OTDs. SA was reported as means and standard deviations, medians and IQRs, and ranges; *p*‐value < 0.05 was considered statistically significant.

For these variables, a receiver operating characteristic (ROC) curve was graphed, and area under the curve (AUC) was used to evaluate whether cartilaginous SA and/or osseous SA can be used as an effective diagnostic test. To establish cutoff values that maximised sensitivity plus one minus specificity, values were selected utilising Youden's Index [37]. The odds ratio of being a PD relative to control based on this proposed diagnostic cutoff value were calculated using logistic regression modelling.

Intraobserver and interobserver reliability were assessed using the intraclass correlation coefficient (ICC). SA measurements were recorded by both raters on 30 randomly selected knees from the study population. To evaluate consistency, the same raters repeated the measurements blindly after a 2‐week interval. Reliability was classified as poor for ICC values below 0.50, moderate for values between 0.50 and 0.74, good for values between 0.75 and 0.90, and excellent for values greater than 0.90 [14].

## RESULTS

**Figure 2:**
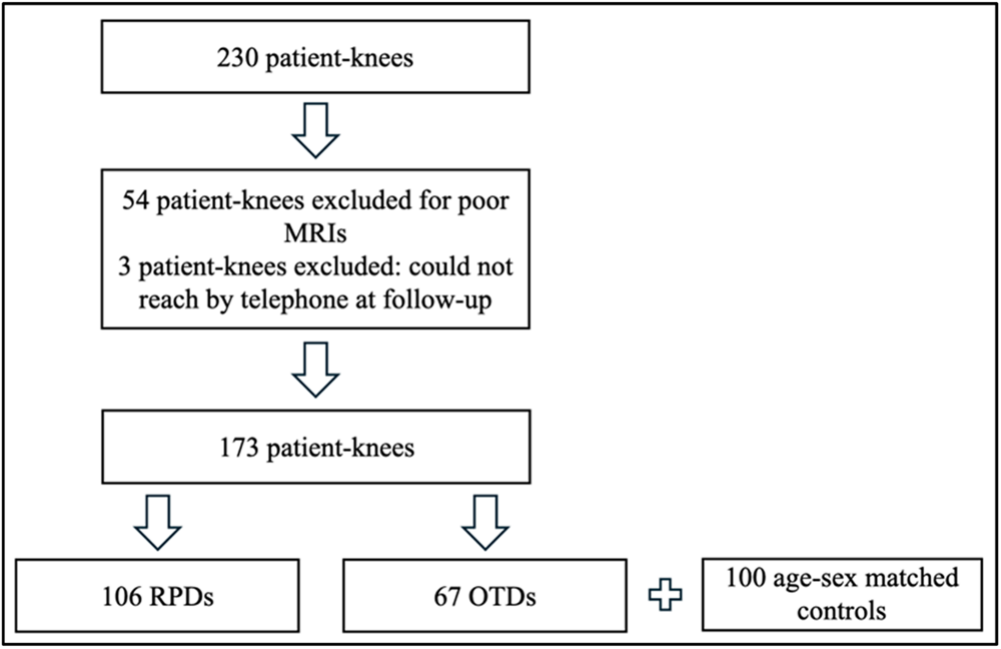
CONSORT chart depicting study cohorts. Created utilising Microsoft PowerPoint.

Of the 230 knees with PD that met inclusion criteria, 57 knees were excluded because of absent or poor MRI studies or because they were lost to follow‐up. The final count of PD knees was 173: the RPD cohort included 106 knees (97 patients; average follow‐up time 5.40 years, range 2.10, 11.43), and the OTD cohort included 67 knees (65 patients; average follow‐up time 4.75 years, range 2.05, 10.40) (Figure 2). There was a total of 100 age and sex frequency matching controls from the internal database of patients with ACL injuries but no history of PD. There was no difference between age at MRI (PD average 15.29 standard deviation 2.45 years, controls 15.56 standard deviation 1.40 years; *p* = 0.25) or gender (PD 58% female, controls 47% female; *p* = 0.13) distributions between cohorts.

**Figure 3:**
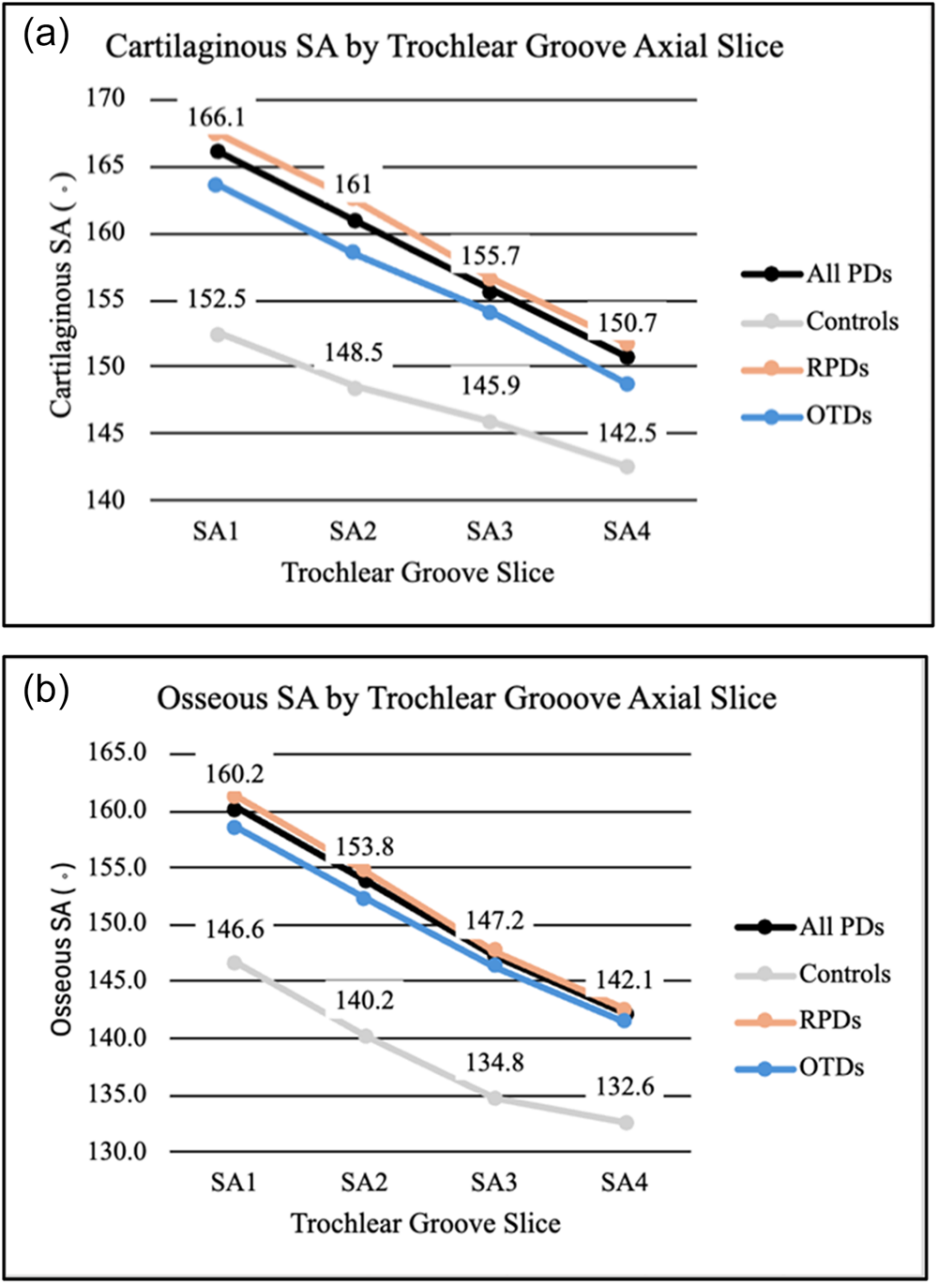
Line graft depicting sample mean SA for cartilaginous (a) and osseous (b) measurements in all PDs, controls, RPDs, and OTDs. Created utilising Microsoft Excel. OTD, one‐time dislocator; PD, patellar dislocator; RPD, recurrent patellar dislocator; SA, sulcus angle; TD, trochlear depth.

When comparing PDs and controls, the mean SA values were greater in patients with PD at all four levels of the trochlear groove for both cartilaginous and osseous measurements (Table 1; *p* < 0.001). The SA (cartilaginous and osseous) was greater at proximal levels of the TG than at more distal locations for both PDs and controls (Figure 3a,b; *p* < 0.001). Cartilaginous measurements were greater than osseous measurements at all levels of the TG in both PD and control cohorts (Figure 3a,b; *p* < 0.001).

**Table 1:** Comparative statistics between study cohorts.

		Control (*N* = 100)	All PD (*N* = 170)	*p*‐value	AUC	RPD (*N* = 106)	OTD (*N* = 64)	*p*‐value
SA1 (c)	Mean	152.5	166.1	*p* < 0.001	0.850	167.5	163.6	*p* = 0.014
	SD	6.8	9.4			10.1	12.4	
	Median	151.6	165.9			166.9	162.8	
	IQR	148.3, 156.7	158.8, 172.7			161.6, 174.5	154.9, 170.0	
	Range	137.8, 171.7	140.3, 201.5			140.3, 189.0	142.4, 201.5	
SA1 (o)	Mean	146.6	160.2	*p* < 0.001	0.870	161.2	158.5	*p* = 0.057
	SD	6.8	11.0			9.0	13.6	
	Median	146.3	159.3			161.5	156.9	
	IQR	143.6, 150.8	153.0, 166.3			154.3, 167.1	150.1, 163.4	
	Range	128.5, 171.1	136.0, 205.5			141.1, 184.5	136, 205.5	
SA2 (c)	Mean	148.5	161.0	*p* < 0.001	0.871	162.5	158.5	*p* = 0.004
	SD	5.7	9.4			8.7	10.0	
	Median	147.6	160.9			161.9	159.0	
	IQR	144.6, 153.0	154.3, 166.4			156.4, 168.5	151.3, 164.9	
	Range	133.7, 162.3	137.9, 185.7			142.5, 180.9	137.9, 185.7	
SA2 (o)	Mean	140.2	153.8	*p* < 0.001	0.872	154.7	152.2	*p* = 0.070
	SD	6.6	10.6			9.1	12.5	
	Median	139.6	153.8			155	150.8	
	IQR	135.2, 145.0	146.4, 159.6			148.0, 160.2	143.7, 157.0	
	Range	126.1, 158.0	133.4, 202.0			133.4, 180.2	135.2, 202	
SA3 (c)	Mean	145.9	155.7	*p* < 0.001	0.835	156.6	154.1	*p *= 0.027
	SD	5.9	8.3			8.3	8.1	
	Median	145.7	155.3			156.6	153.7	
	IQR	141.8, 150.2	150.0, 160.4			150.8, 162.0	148.4, 158.8	
	Range	131.0, 163.9	134.0, 179.0			137.6, 179.0	13.0, 174.4	
SA3 (o)	Mean	134.8	147.2	*p* < 0.001	0.867	147.7	146.3	*p *= 0.185
	SD	6.1	9.3			9.3	9.3	
	Median	134.9	146.7			147.3	145.9	
	IQR	130.5, 138	141.6, 152.8			142.0, 153.4	140.0, 150.7	
	Range	122.5, 152.2	125.9, 173.4			125.9, 172.8	128.7, 173.4	
SA4 (c)	Mean	142.5	150.7	*p* < 0.001	0.782	151.7	148.7	*p* = 0.007
	SD	6.5	7.9			8.3	6.8	
	Median	143.0	150.2			150.5	148.0	
	IQR	138.2, 147.5	144.8, 155.7			145.2, 156.9	144.4, 153.2	
	Range	127.5, 159.7	132.3, 174.3			136.7, 174.3	132.3, 161.1	
SA4 (o)	Mean	132.6	142.1	*p* < 0.001	0.836	142.5	141.4	*p *= 0.175
	SD	6.2	7.9			7.6	8.4	
	Median	132.7	141.9			142.35	140.3	
	IQR	127.8, 136.3	136.9, 147.3			137.4, 147.5	135.7, 147.0	
	Range	119.1, 154.4	121.4, 164.5			121.4, 164.5	124.5, 163.0	

*Note*: All SA measurement units are degrees.

Abbreviations: (c), cartilaginous; (o), osseous; AUC, area under the curve; OTD, one‐time dislocator; ROC, receiver operating characteristics; RPD, recurrent patellar dislocator; SA, sulcus angle.

When comparing RPDs and OTDs, the mean SA values were greater in patients with RPD at all four levels of the trochlear groove for cartilaginous measurements; however, no statistically significant difference was found between these cohorts for osseous measurements (Table 1).

**Figure 4:**
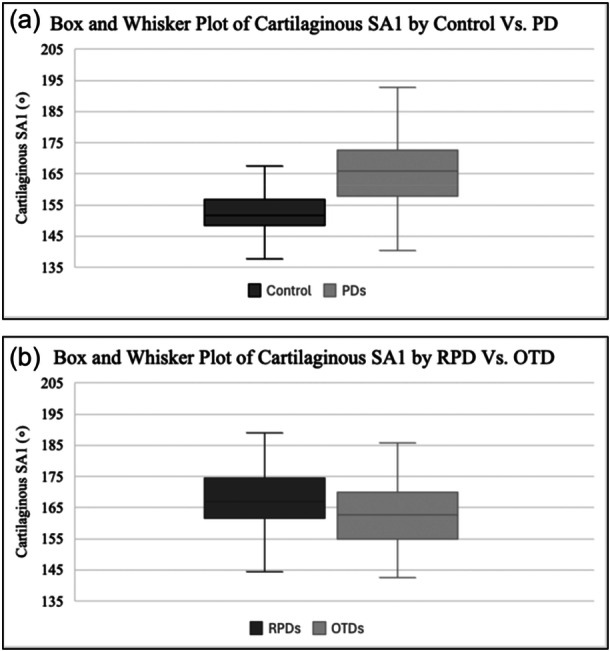
Box and Whisker plots comparing the distribution of cartilaginous SA1 between controls versus PDs (a) and between RPDs and OTDs (b). OTD, one‐time dislocator; PD, patellar dislocator; RPD, recurrent patellar dislocator; SA, sulcus angle.

AUC values determined from ROC curves indicate excellent or good discrimination between PDs and controls for cartilaginous SA1–4 and osseous SA1–4 (Table 1). Diagnostic cutoff values for TD are greater for more proximal than distal levels and along cartilaginous compared to osseous measurements (Table 2). Based on the simple boxplots (Figure 4a,b), there is no overlap between the IQRs of controls and PDs, suggesting a meaningful cutoff value can be identified. However, there is substantial overlap between the IQRs of RPDs and OTDs, suggesting it would be difficult to identify a statistically meaningful cutoff value.

**Table 2:** Odds ratio of being a PD versus control for associated diagnostic cutoff values.

		Cutoff value (°)	Sensitivity	Specificity	Odds ratio (95% CI)
SA1 (c)	Control vs. PD	159.6	0.735	0.857	17.1 (8.7, 33.6)
SA1 (o)	Control vs. PD	153.1	0.753	0.888	23.4 (11.4, 47.8)
SA2 (c)	Control vs. PD	153.8	0.776	0.827	16.0 (8.5, 30.2)
SA2 (o)	Control vs. PD	148.0	0.700	0.929	30.3 (13.2, 70.0)
SA3 (c)	Control vs. PD	152.5	0.665	0.888	15.7 (7.7, 31.7)
SA3 (o)	Control vs. PD	141.6	0.753	0.878	21.8 (10.9, 43.9)
SA4 (c)	Control vs. PD	148.1	0.612	0.806	6.6 (3.6, 11.8)
SA4 (o)	Control vs. PD	137.4	0.718	0.827	11.8 (6.3, 21.9)

*Note*: Intraobserver ICCs were excellent for SA1‐4 (cartilaginous, c & osseous, o) (Table 3). Interobserever ICCs were excellent for SA1 (c), SA2 (c), SA4 (c), SA1 (o), and SA2 (o), and good for SA3 (c) and SA3 (o), and SA4 (o) (Table 4).

Abbreviations: (c), cartilaginous; (o), osseous; CI, confidence interval; ICC, intraclass correlation coefficient; PD, patellar dislocator.

**Table 3:** Intraobserver reliability as determined by the intraclass correlation coefficient.

	SA1 (c)	SA1 (o)	SA2 (c)	SA2 (o)	SA3 (c)	SA3 (o)	SA4 (c)	SA4 (o)
Rater 1	0.98 (0.96, 0.99)	0.97 (0.94, 0.99)	0.93 (0.85, 0.97)	0.97 (0.93, 0.98)	0.98 (0.97, 0.99)	0.98 (0.97, 0.99)	0.96 (0.91, 0.98)	0.98 (0.96, 0.99)
Rater 2	0.94 (0.88, 0.97)	0.94 (0.87, 0.97)	0.94 (0.87, 0.97)	0.89 (0.77, 0.95)	0.97 (0.93, 0.98)	0.95 (0.89, 0.98)	0.94 (0.88, 0.97)	0.93 (0.84, 0.97)

*Note*: Data in parentheses represents 95% CI.

Abbreviations: (c), cartilaginous; (o), osseous; CI, confidence interval; SA, sulcus angle.

**Table 4:** Interobserver reliability as determined by the interclass correlation coefficient.

SA1 (c)	SA1 (o)	SA2 (c)	SA2 (o)	SA3 (c)	SA3 (o)	SA4 (c)	SA4 (o)
0.91 (0.35, 0.97)	0.91 (0.38, 0.97)	0.90 (0.44, 0.97)	0.93 (0.41, 0.98)	0.85 (−0.02, 0.96)	0.83 (−0.11, 0.95)	0.94 (0.88, 0.97)	0.77 (0.13, 0.93)

## DISCUSSION

The SA is a highly useful measure of TD and diagnostic test for discriminating between PDs and controls. It is significantly greater in PD patients compared to patients without PD in proximal and distal sections as well as in cartilaginous and osseous measurements. To identify whether a SA measurement is dysplastic, the axial level and surface being assessed must be considered to apply the appropriate cutoff value. Several trends in the analysis of cutoff values were observed. There is no universal cutoff value for SA, and instead, this value differs between cartilaginous and osseous surfaces and by TG location. The cartilaginous diagnostic cutoff is higher than the osseous cutoff at all TG levels. The optimal cutoff decreases proximally to distally. Excellent or good reliability for all measures of cartilaginous and osseous SA was observed. Importantly, only cartilaginous SA measurements discriminate between RPDs and OTDs; there is no statistically significant difference in osseous SA values between these cohorts at any of the four levels of the TG.

In this study, the range of optimal cutoff values was between 149° and 160° for cartilaginous measurements and between 137° and 153° for osseous measurements. This range aligns closely with several studies that suggested values ranging from 144° to 160° [4, 16, 29, 31]. While this range could be perceived as too wide to be clinically useful, these results support that there is in fact a wide range of acceptable pathological cutoffs that vary based on the MRI axial section and the surface being evaluated. Additionally, despite this range, several MRI‐based studies have reported excellent reliability and clinical utility of SA; one study identified an AUC of 0.82 for measuring cartilaginous SA and 0.79 for osseous SA measurements [3, 4, 16, 31, 34].

Measuring four consecutive axial images of the TG allowed for more comprehensive understanding of the trochlear morphology. Measures of SA are consistently highest proximally, on cartilaginous surfaces, and among those with PD (Figure 3). These figures demonstrate that the sample mean difference between dislocators and controls is greater proximally (PD cartilaginous SA1 minus control cartilaginous SA1 equals 13.6°, PD osseous SA1 minus control osseous SA1 equals 13.6°) than it is distally (RPD cartilaginous SA4 minus control cartilaginous SA4 equals 8.2°, RPD osseous SA4 minus control osseous SA4 equals 9.5°) (*p* < 0.001). Thus, while TD may be observed throughout the TG, these findings verify prior studies that it is most pronounced more proximally [12, 18].

Multiple previous studies have described techniques to consistently identify one axial image within the TG to measure SA. Pfirrmann et al. established that the TG could be identified 3 cm above the femorotibial joint space [19]. Ambra et al. used an oblique trochlear MRI view to assess trochlear morphology at different levels (25%, 50%, and 75% of the trochlear length) [1]. Tanaka et al. used the Roman Arch appearance of the intercondylar notch to compare anatomy across knees [31]. Finally, Vranken et al. recorded TD at 5 or 1 cm from the superior margin of the medial trochlear facet [36]. While these studies found that measurement height impacts the value of measurements, a consensus method for identifying the proximal TG has not been identified. Additionally, these studies consider just one axial image even though the typical trochlear length is 2 cm, suggesting that there are several relevant MRI axial images (which are typically captured 3 mm apart of the TG) [4, 35]. Measuring four consecutive axial images, as was done in this study, provides a more thorough assessment of the extent of TD.

## LIMITATIONS

Certain limitations warrant consideration in this study. In total, 57 patients were excluded. MRI image quality constraints led to the exclusion of 44 knees from analysis. Additionally, three patients initially classified as OTDs through chart review were excluded because we were unable to confirm their status via telephone follow‐up. The retrospective design introduces potential selection bias that may impact the strength of the findings. Despite these limitations, the final sample size remained sufficient to support the statistical conclusions presented.

The use of just two raters, a medical student and an orthopaedic surgery resident, represents a potential limitation. Additionally, conducting the intra‐observer reliability analyses just 2 weeks apart, as opposed to 4 or more weeks, could introduce bias.

## CONCLUSIONS

SA was greater in PDs than controls at all four levels in the TG for both cartilaginous and osseous measurements. Cartilaginous SA was greater among RPDs than OTDs at all levels; however, osseous SA was not different between cohorts. The diagnostic cutoff of dysplastic SA differed by axial level and surface.

## AUTHOR CONTRIBUTIONS

**Benjamin J. Levy**: Conceptualisation; methodology; writing—review and editing. **Edina Gjonbalaj**: Resources. **Eric D. Fornari**: Supervision. **Jacob Schulz**: Supervision. **Jason D. Brenner**: Conceptualisation; methodology; formal analysis and investigation; writing—original draft preparation. **Leila Mehraban Alvandi**: Resources. **Mauricio Drummond Junior**: Conceptualisation; methodology; formal analysis and investigation; writing—review and editing. **Steven M. Henick**: Methodology; formal analysis and investigation; writing—review and editing. **Yungtai Lo**: Resources.

## CONFLICTS OF INTEREST STATEMENT

Jacob Schulz is a paid consultant for Johnson & Johnson and Orthopediatrics. The remaining authors declare no conflict of interest.

## ETHICS STATEMENT

This retrospective chart review study involving human participants was in accordance with the ethical standards of the institutional and national research committee and with the 1964 Helsinki Declaration and its later amendments or comparable ethical standards. The Human Investigation Committee (IRB) of Montefiore Medical Center (IRB: 2016‐6534) approved this study.

## Data Availability

The data that support the findings of this study are available from the corresponding author upon reasonable request.
